# A tale of ‘politics and stars aligning’: analysing the sustainability of scaled up digital tools for front-line health workers in India

**DOI:** 10.1136/bmjgh-2021-005041

**Published:** 2021-07-21

**Authors:** Neha S Singh, Kerry Scott, Asha George, Amnesty Elizabeth LeFevre, Rajani Ved

**Affiliations:** 1 Department of Global Health and Development, Faculty of Public Health and Policy, London School of Hygiene and Tropical Medicine, London, UK; 2 Department of International Health, Johns Hopkins University Bloomberg School of Public Health, Baltimore, Maryland, USA; 3 School of Public Health, University of the Western Cape, Faculty of Community and Health Sciences, Cape Town, Western Province, South Africa; 4 School of Public Health and Family Medicine, University of Cape Town, Rondebosch, Western Cape, South Africa; 5 National Health Systems Resource Centre, New Delhi, Delhi, India

**Keywords:** health policy, health systems, qualitative study, public Health

## Abstract

**Introduction:**

India has become a lighthouse for large-scale digital innovation in the health sector, particularly for front-line health workers (FLHWs). However, among scaled digital health solutions, ensuring sustainability remains elusive. This study explores the factors underpinning scale-up of digital health solutions for FLHWs in India, and the potential implications of these factors for sustainability.

**Methods:**

We assessed five FLHW digital tools scaled at the national and/or state level in India. We conducted in-depth interviews with implementers, technology and technical partners (n=11); senior government stakeholders (n=5); funders (n=1) and evaluators/academics (n=3). Emergent themes were grouped according to a broader framework that considered the (1) digital solution; (2) actors; (3) processes and (4) context.

**Results:**

The scale-up of digital solutions was facilitated by their perceived value, bounded adaptability, support from government champions, cultivation of networks, sustained leadership and formative research to support fit with the context and population. However, once scaled, embedding digital health solutions into the fabric of the health system was hampered by challenges related to transitioning management and ownership to government partners; overcoming government procurement hurdles; and establishing committed funding streams in government budgets. Strong data governance, continued engagement with FLHWs and building a robust evidence base, while identified in the literature as critical for sustainability, did not feature strongly among respondents. Sustainability may be less elusive once there is more consensus around the roles played between national and state government actors, implementing and technical partners and donors.

**Conclusion:**

The use of digital tools by FLHWs offers much promise for improving service delivery and health outcomes in India. However, the pathway to sustainability is bespoke to each programme and should be planned from the outset by investing in people, relationships and service delivery adjustments to navigate the challenges involved given the dynamic nature of digital tools in complex health systems.

**Key questions**

**What is already known?**

- Hundreds of digital health interventions have been piloted in low-income and middle-income countries, though few have been successfully scaled.
- Among those that have progressed beyond pilot initiatives to attain scale, efforts to achieve sustainability remains elusive, particularly with regard to integration into routine health services delivery, independence from donor funding, interoperability and governance.

**What are the new findings?**

- To successfully sustain a scaled up digital tool, it is imperative for all stakeholders, in particular governments and donors, to have an entire supportive ecosystem in place that addresses the dynamics between aspects of the digital solution, actor relationships, implementation processes and key contextual factors, with strong government leadership to align all these pieces.

**What do the new findings imply?**

- Our findings challenge the notion that digital health programmes progress linearly from pilot to scale to sustainability, and instead explore the elements and tensions between scale and sustainability for digital health.
- With significant resources spent each year on digital health solutions that are never scaled or sustained, it is imperative that we build the evidence base on factors that lead to success in sustaining innovations in the digital health space.

## Introduction

Throughout the last decade, over 600 digital health pilot strategies and programmes have been introduced globally.^1^ Despite the proliferation of digital health programmes, evidence on their effectiveness is limited.^2–4^ Moreover, governments have found it challenging to select, scale-up, and integrate solutions into existing national systems.^5^

Scale-up refers to the increase of a programme’s reach from a pilot population or experimental region to greater numbers of people over wider geographical areas.^6^ Studies and frameworks examining scaling up have emerged over time with considerable commonality.^7–10^ Sustainability of a programme is the ‘capability of being maintained at a certain rate or level.’^11^ When applied to health programmes and policies, the concept of sustainability involves the continuation of inputs (eg, funding or programme activities), outputs (eg, health benefits) and a process of adaption in response to emerging needs of the system.^12^ It is an emergent field of enquiry, with numerable frameworks from different disciplines and noted challenges in assessment.^10 12–16^

**Figure 1:**
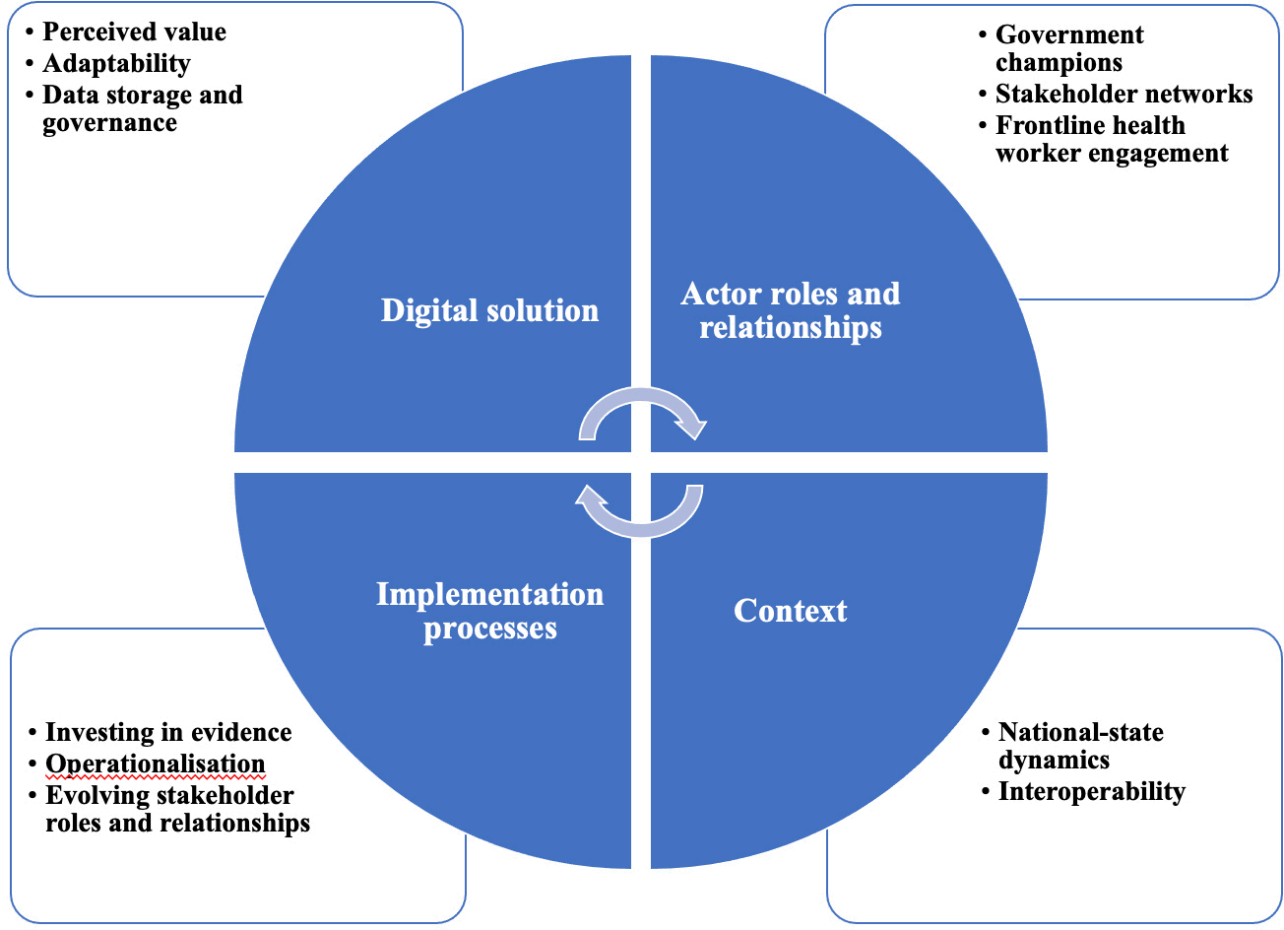
Conceptual framework for evaluating the scale-up and sustainability of digital solutions for front-line health workers.

To date, only select digital health programmes for front-line health workers (FLHWs) have scaled,^17 18^ including a few in India.^19–21^ The successful scale-up of these programmes raises important questions about the micro (eg, negotiations between individuals), meso (organisational processes and systems) and macro (national policy and wider context) level factors that underpin decision making on which initiatives get adopted and with what evidence. Major factors influencing scale-up have been synthesised to create our conceptual framework (discussed below, figure 1).^10 22–24^ The features that influence sustainability overlap considerably with those for scale-up, but shift focus from the intervention itself to the health system’s ability to plan, organise, adapt and communicate, and the broader fiscal and political environment.^25 26^

To date, very few studies^27 28^ have aimed to understand processes underpinning scaled digital health tools in India or elsewhere to reflect on sustainability. Emerging literature, largely from high-income settings, sheds light on the complex and multi-dimensional nature of scaling digital solutions for health,^13 29–31^ as well as how the social, political and historical contexts where digital tools are implemented plays a key role in how solutions are embedded, adapted and potentially sustained within a health system.^32 33^

This study aims to answer ‘Why have some digital health programmes scaled, and others not, and what are the implications for sustainability in India?’. To understand the factors that underpin the scale-up and sustainability of different typologies of digital health solutions in India, we examine five FLHW digital tools ranging from direct-to-FLHW programmes to real-time data capture and decision-support tools. This analysis will benefit actors involved in the development of digital tools for FLHWs in India and other settings by illuminating the multifaceted inputs and processes involved in scale-up and by providing a framework for thinking about how the scale-up process can support or hinder the tool’s chances of sustainability. Drawing case studies from India is particularly fruitful because it is the site of several of the world’s largest digital programmes for FLHWs and one of the few low-income or middle-income countries that appear to be sustaining these tools.

## Methods

### Conceptual framework

We developed a conceptual framework to study what shapes scale-up and sustainability of digital tools for FLHWs (figure 1), reflecting broader literature on the subject.^6 8 10 13 31 34–37^

Our framework was adapted from the following existing conceptual frameworks by taking into account variables relevant to the implementation of digital tools in resource-constrained settings: (1) Greenhalgh *et al*’s framework taking into account complexity of scaling-up technology-supported programmes^13^; (2) Gericke *et al*’s framework which has been applied to assess the scale-up of mHealth innovations in Malawi and Zambia^31^; (3) Spicer *et al*’s framework based on studying scalable health innovations’ attributes in Ethiopia, India and Nigeria^10^ and (4) Gilson and Walt’s policy triangle.^38^ Doing so enabled us to simplify relevant variables into four themes: (1) digital solution characteristics, (2) actor roles and relationships, (3) implementation processes and (4) context. While drawing from existing frameworks for scaling and sustaining health interventions more generally, our work particularly takes into account specific ‘hardware’(eg, cloud storage) and ‘software’ (eg, technological partnerships) required to scale and sustain digital health solutions.

### Study setting

In 2011, India’s population was 1.2 billion, with nearly three-quarters (74%) being literate.^39^ Mobile phone access in India has rapidly increased, with the 2015–2016 National Family Health Survey (NHFS) reporting 90% of households having access to mobile phones. However, less than half of women surveyed (46%) have access to mobile phones.^40^

India has a federal health system structure, where health is a state subject but national government defines key strategies and programmes.^41^ For example, the Ministry of Health and Family Welfare (MoHFW) is responsible for national programmes for health and family welfare, prevention and control of communicable diseases, promotion of traditional and indigenous systems of medicines, and setting standards and guidelines, which state governments can adapt. Additionally, The Ministry of Women and Child Development (MoWCD) is responsible, among other programmes, for implementing the Integrated Child Development Services (ICDS) programme, in collaboration with the MoHFW, which provides a package of services including supplementary nutrition, immunisation, health check-ups and referral services, and preschool education.

The 2015–2016 NHFS reported that utilisation of key maternal and newborn health services are variable and characterised by breaks in the continuity of care. While most women (83%) attend at least one antenatal care visit, only half (51%) receive the recommended four visits. Despite high skilled birth attendance (81%), provision of postnatal care is uneven for mothers and newborns; with only 65% of mothers and 27% of newborns receiving postnatal care within 2 days of birth.^40^

### Cases

In this study, programmes were considered to be sufficiently scaled up to serve as case studies if they were reaching a large proportion of eligible FLHWs across at least one state in India.

Case studies of varying complexity were selected based on three features. First, our cases showcase a range of technical features such as data capture, decision-support, direct-to-FLHW health information messages. Second, they are at different levels of maturity in terms of scale and sustainability, which enabled us to explore differences in their experiences in scaling and varying levels of success in being sustained. And third, they are geographically diverse, enabling an examination of contrasting Indian governmental state capacities. Our cases and respective digital tools are as follows: (1) TECHO +in Gujarat; (2) Mobile Academy (MA) in Gujarat and Madhya Pradesh; (3) Anmol in Madhya Pradesh and at national level; (4) the non-communicable diseases (NCDs) App at the national level and (5) Common Application Software (CAS) at the national level. Table 1 compares and contrasts each case study in terms of their components and functions, actors involved and coverage.

**Table 1:** Case study overview

Case study (inception year)	Components/functions	Actors	Coverage
Mobile Academy for India’s Accredited Social Health Activist (ASHA) community health workers* (piloted from 2012, scaled in 2016)	Phone-in interactive voice response (IVR) mobile refresher training designed to improve FLHWs interpersonal communication skills on preventative reproductive, maternal, newborn and child health (RMNCH) behaviours. 44 short (~2.5 minute) pre-recorded audio lessons; total course length 240 minutes. 44 yes/no, multiple choice quiz questions. MOTECH system which is interoperable with and ingests mobile phone numbers of ASHAs from Maternal and Child Tracking System (MCTS)/Reproductive Child Health (RCH; validation checks help to bolster MCTS/RCH data quality. *Mobile Academy was designed to work together with two ‘sister’ programmes, also developed by BBC Media Action and initially implemented in Bihar. Kilkari, a direct-to-FLHW health message programme which delivers prerecorded audio health information messages weekly to pregnant and postpartum women and has been scaled across 13 states nationally. Mobile Kunji, an audiovisual job aid to help ASHAs communicate with community members. ASHAs use a deck of 40 colour-coded cards with illustrations, and access supporting audio by dialling into an IVR service. Mobile Kunji has not been scaled up beyond the state of Bihar, where it has been adopted by the Bihar State Health Society.	Technology development: Beehyv, Grameen Foundation, Dimagi Technical development and implementation: BBC Media Action Funders: BMGF, USAID, the Barr Foundation Programme support and management: Government of India’s MoHFW; National Informatics Centre (NIC); Price Waterhouse Coopers Telecommunications, IVR, hosting and data centre: Reliance Communications Limited, Railtel, IMI Mobile.	Implemented in 13 of 29 states. Around 267 000 ASHAs, 43% of all ASHAs registered in the government’s databases, had started the course (as of March 2019). 180 500 (30%) of all ASHAs registered, had completed it as of March 2019.
ANMOL for auxiliary nurse midwives (ANMs), India’s front-line subnursing cadre (2017)	Android-based tablet application for ANMs that supports data capture and service delivery planning for health and nutrition services to pregnant women, mothers and children <12 months. Preloaded audio and video files used to counsel women and couples on subjects like high-risk pregnancies, immunisation and family planning. The tablets maintain an auto-generated list of pending tasks. Interoperability with the RCH database—it feeds data captured by ANMs into the RCH database Comprehensive dashboards display reports on data captured.	Technology development: Dhanush Infotech Technical development and implementation: Dhanush Infotech with other technology partners Funders: Unicef and Ministry of Health and Family Welfare (MoHFW) Programme support and management: Government of India’s MoHFW, NIC.	Implemented in 9 of 29 states 50 000 ANMs of 293 000 in India were using the tool as of early 2018
NCD App for ANMs (piloted from 2018, scaled in 2021)	A comprehensive primary healthcare platform (CPHP) that supports technical interoperability with the MoHFW’s MCTS, RCH and other electronic health information systems. An Android application on a tablet that sits on the CPHP and supports ANMs in conducting NCD screening and management for all adults over 30 years of age.	Technology and technical development: Dell EMC Implementation: Tata Trusts Funders: Dell EMC, Government of India Programme support and management: Government of India’s MoHFW.	Implemented in 26 of 29 states Almost 100 000 tablets in the field running the NCD app Nearly 20 000 ANMs have logged in over the last 30 days (as of Oct 2019)
TECHO+ (Technology Enabled Community Health Operations) for ANMs and ASHAs (piloted from 2013; scaled in 2017)	Data capture, decision-support and scheduling android application for mobile phones used by ASHAs and ANMs to deliver to deliver health and nutrition services to pregnant women, mothers and children <12 months. Decision support in form of digital checklist to encourage adherence to protocols during home visits. Scheduling and activity planning in form of reminder to ANMs to plan for village health and nutrition day. Longitudinal, digital tracking of pregnant women and infants’ health status and services. NCDs, nutrition, developmental delays, communicable diseases, and mental health modules are being added to achieve comprehensive primary healthcare in alignment of Ayushman Bharat. Targeted client communication using multimedia to transmit targeted health information and improve counselling for behaviour change communication.	Technology development: Argusoft India Ltd., Gandhinagar Implementation: SEWA Rural, Jhagadia Funders: Government of Gujarat, UNICEF (research funding from Indian Council of Medical Research, WHO and MacArthur Foundation) Programme support and management: GVK EMRI, Government of Gujarat	90% of Gujarat’s population enrolled in TeCHO+ (as of Feb 2019) 100% of all pregnant women and under five children enrolled Gujarat Government health department and National Health Mission gave 11 000 smartphones and data plans to all ANMs in 2018
Common Application Software (CAS) for Anganwadi workers (AWWs), India’s community pre-school and nutrition supplementation workers (2018)	Data capture and decision-support android application for mobile phones used by AWW and their supervisors to deliver health and nutrition services to pregnant women, mothers and children <12 months. The CAS AWW app replaces 10 of the 11 paper-based registers AWWs used to maintain and consists of 8 modules: household management, home visit scheduler, daily nutrition, growth monitoring, take-home rations, due list, Anganwadi centre management and monthly progress report. The CAS supervisor app providers supervisors with a checklist which allows them to identify how the AWWs in 10–20 Anganwadi centres are performing, and provides data to inform discussions at monthly sector meetings. CAS web-enabled dashboard allows real-time monitoring by Integrated Child Development Services (ICDS) officials CAS is supported by the CommCare, an open access technology platform. CommCare is not interoperable with the MoHFW’s RCH or MCTS databases but creates its own database which serves as a data repository for the Ministry of Women and Child Development.	Technology development and implementation: Dimagi Technical development and implementation: BMGF Funding: BMGF, MoWCD	Implemented in 24 of 29 states. ~354 000 Anganwadi Centres using ICDS-CAS (as of June 2019).

FLHW, front-line health workers; MoWCD, Ministry of Women and Child Development; NCD, non-communicable diseases.

### Data collection

We conducted semistructured in-depth interviews with respondents identified using investigator contacts and snowball sampling in person in New Delhi, Bangalore, Bhopal and Ahmedabad from May to October 2019, and remotely using Zoom software from July to October 2020. Respondents were sampled from the following categories: technology partners, implementers and technical partners (n=11); senior government stakeholders who had played key roles in commissioning, scaling and/sustaining the digital tools (n=5); funders (n=1) and evaluators/academics (n=3) (table 2). Our sample size was limited due to COVID-19 pandemic starting in the middle of our study, which impacted respondents’ availability, as well as our ability to follow up with them in person. However, several respondents (n=7) had in-depth knowledge of multiple cases: MA (n=8); TECHO+ (n=5); ANMOL (n=5); NCD app (n=4); CAS (n=9).

**Table 2:** Key informants Interviewed by respondent type with knowledge of specific case studies

Category	Cases represented*	N
Technology partner/implementers/technical partner	Mobile Academy, TECHO+, Anmol, NCD app, CAS	11†
Government	Mobile Academy, TECHO+, Anmol, NCD app, CAS	5
Funder/donor	Mobile Academy, CAS	1
Evaluator/academic	TECHO+, CAS	3†
**Total**		**19**

*Respondents across categories had knowledge of multiple cases: Mobile Academy (n=8); TECHO+ (n=5); ANMOL (n=5); NCD app (n=4); CAS (n=9).

†One respondent (KI07) is classified as both a technology partner/implementer and academic.

NCD, non-communicable diseases.

Research began by introducing the participant to the study, providing them with an information sheet and consent form for the study, and obtaining and recording verbal consent only after giving them sufficient time to consider whether or not to participate in the research and answering any questions they may have. Interviews were conducted in English using a semistructured interview guide covering domains in the conceptual framework (figure 1). Interviews lasted approximately 1–1.5 hours. At the end of each interview, respondents were asked if they knew anyone with experience relevant to the subject of our research. Researchers took detailed notes and audio recorded each interview, which was transcribed for analysis.

### Data analysis

Analysis involved the following stages: NSS and KS systematically coded the interview transcripts using Dedoose software, adopting a framework approach whereby a priori and emerging themes were applied. KS prepared a detailed synthesis report that summarised findings by emerging themes and NSS, KS, AG and AEL participated in an analysis workshop, where emerging findings were reviewed and jointly agreed and the conceptual framework for the study was revised to reflect the study’s findings. Data were then revisited using the revised conceptual framework with the paper drafted by NSS with inputs from KS and reviewed by all authors to confirm the findings are accurately and coherently presented.

We followed Noble and Smith’s recommended steps to enhance the validity and reliability of qualitative data collection and analysis, including accounting for personal biases, frequent communication with all researchers in the study team and ongoing critical reflection of methods to ensure sufficient depth and relevance of data collection and analysis.^42^ We operationalised these steps through convening planning and debrief meetings before and after each KI to: revisit the interview guide and focus our interview strategy, discuss the detailed interview notes and high level summary comments, and continually re-evaluate our impressions and interpretations of responses to ensure that personal bias was minimised. A preliminary analysis report was also reviewed by the team and discussed at length before proceeding with drafting the manuscript.

### Patient and public involvement statement

Given the nature of our study—a high-level policy analysis—it was not appropriate to involve patients or the public in our research.

## Results

We now apply the framework to our cases, focusing on the potential impact of salient features within the domains (digital solution, actor roles and relationships, implementation processes and context) on the digital health solution’s scale-up, and the implications of these aspects of scale-up on the sustainability of these solutions going forward.

### Digital solution

Here, we report characteristics of the digital solution that affected scale and are likely to affect sustainability: their perceived value to all stakeholders, bounded adaptability and data governance.

#### Perceived value

Findings suggest that the perceived value of these digital health solutions arose from responding to the needs of various actors. MA was valued as an affordable, standardised and logistically simple mechanism to refresh Accredited Social Health Activist’s knowledge gained from face to face trainings and fill in knowledge gaps. For the range of digital health solutions focused on FLHW data capture, workflow planning and/or decision support (ANMOL, TECHO+, CAS, NCD app), respondents reported valuing the potential to streamline data collection systems and improve the timeliness, quality, accessibility and use of data.

While fulfilling a clear health system need enabled these digital tools to scale, respondents felt that sustainability hinged on tools becoming inherent to effective service delivery. Respondents argued that this underlined the success of one state-level solution (KI14, evaluator/academic), while another speculated that sustainability was not guaranteed because the transformative potential for service delivery was yet to be grasped.

I think to some degree, yeah, sustainability is a pipe dream. … Like, digital is viewed as an IT product, it’s viewed as a data collection mechanism to get you reports. They don't use the reports to do anything useful. So, they're not buying the digital health technology … because it improves outcomes… (KI16, technology partner)

#### Adaptability

Most key informants identified adaptability as a key enabling feature for scaling up, but one that was bounded by certain technical nuances. They distinguished between software that is hardcoded versus configurable. In order to make changes to hardcoded software a senior engineer is needed to manually rewrite the code; a process which can be time consuming and expensive. By comparison, configurable software allows technology partners to quickly and easily configure different parameters without editing the source code. They specified that configurability was a more scalable approach and seen as essential for accommodating differences in state-level health programmes and priorities. For instance, MA was configurable because new curriculums with different numbers of chapters and quizzes could be easily set up using the existing software and infrastructure (KI01, technical partner). However, informants identified that configurability should be carefully managed and permissions based because allowing states to customise data fields would hamper implementation and impede efforts to aggregate and compare data.

In addition to adaptability, informants noted the importance of extensibility—defined as the ability of systems to extend to and accommodate new users or geographic areas. Extensibility was driven by infrastructure and telecommunications connectivity choices. For example, an interactive voice response system is more extensible if it can quickly and easily scale to handle spikes in call traffic and then descale when the spikes have subsided.

#### Data storage and governance

Although respondents agreed that data storage and data governance domains (access, privacy and consent) are key considerations for any digital tool, they did not cite them as a key factor contributing to scale or sustainability. Data storage was mentioned as closely tied to scale-up was the use of high-quality cloud-based data storage rather than physical data storage infrastructure. Government resistance to cloud-based systems, which partially stemmed from concern about data being housed outside the country and ‘exposed’ on the cloud (KI04, technological partner), was reported to be rapidly declining as India-based cloud systems have become available and demonstrated their capacity.

Data governance domains including consent, privacy and data protection were not cited as considerations when scaling or sustaining these digital tools. A respondent speculated that some stakeholders viewed data protection as ‘a luxury for wealthy consumers and that it’s a barrier to saving lives, and it’s totally unrealistic for illiterate poor people who can’t, who have no idea what data even is’ (KI01, technology partner). Low prioritisation of data governance domains had resulted in poor consent processes at the front lines, wherein FLHWs were ‘not really’ taking proper informed consent when collecting data using digital tools (KI10, government). Another respondent worried that failure to invest in consent processes could someday undermine sustainability by undermining public trust:

Explaining these [data protection] concepts to poor, illiterate people is super challenging…[but] if you don't engage and take consent to take people’s data, you have a situation of distrust. (KI01, technology partner)

### Actor roles and relationships

In this section, we consider the government champions, networks and sustained leadership, and engagement with FLHWs, including the relationships involved in developing, implementing and scaling these digital tools that are likely to affect sustainability.

#### Government champions

Champions in influential government ministerial positions in MoHFW and MoWCD and at state government level were vital to the scale-up of all digital tools studied (KI14, evaluator/academic). For example, CAS was made possible by having ‘the right guy for the job’ in government (KI06, donor) who was ‘able to push through all the bureaucratic kind of machinery that was required and to make sure that it (CAS) was moving’ (KI17, technology partner).

Communication between state government actors was an important enabler for scaling MA and the NCD app. In these cases, the programme was first implemented in one state with support from senior actors in that state’s government. These actors went on to praise the programme during interactions with their counterparts in other states, which created champions for the programmes in other states.

#### Stakeholder networks

While a powerful government champion can push a programme forward and approve the decision to scale, sustainability required deeper relationships ‘so it can go all the way down to the people who are actually making things happen’ (KI02, technical partner) and broader buy-in to ensure that the programme continues—especially when individual leaders inevitably move on (KI14, evaluator/academic).

Implementing partners, including non-government organisations, technologists and donors invested heavily in building and sustaining relationships, particularly with bureaucrats at different levels of government (KI14, evaluator/academic). For example, respondents from the technical agency that developed MA described ‘walking the corridors of the National Health Mission’ over several months to build trust (KI02, technical partner).

Beyond government, all our cases showcase the importance of committed, sustained teams and strong leaders at donor, technology, technical, and implementing organisations.

It’s easy to sort of keep looking externally but even internally, if something had happened and our key, you know, drivers of the project internally had left or were involved in something else and therefore not giving this so much time we wouldn't have had the kind of success we had. (KI02, technical partner)

#### FLHW engagement

While digital tools are meant for FLHWs who ultimately determine their use, several respondents noted that FLHWS were not involved in decision making related to scale-up, beyond participating in implementation and research directed by others. As all the tools were required—not optional—for FLHWs, high uptake cannot be considered a proxy for FLHW endorsement. However, a respondent noted that ‘in the end… the true mark of sustainability [of these digital tools] will be that this is something that gets routinised into the day-to-day work of the health worker themselves so they value it, that they demand it, that if it breaks or crashes or does not work that they want it fixed because they find it so valuable’ (KI14, evaluator/academic).

### Implementation processes

In this section, we examine the process underpinning the successful scale-up of these cases, summarised as investments in research evidence, operationalisation considerations and balancing actor roles.

#### Investing in evidence

##### Formative work to support fit with context and population

The cases underwent diverse development processes with implications for their fit with the context and population. While some underwent extensive user testing and embedded development (MA, NCD app, TECHO+), others were first developed by technology partners with specifications/data capture features stipulated, and then adjusted when implemented (ANMOL).

For instance, the NCD app arose from an embedded development process, wherein app developers spent 1.5 years working in a primary health centre ‘lab’ with a small group of FLHWs to develop a locally appropriate digital tool (KI03, technical partner). In contrast, some of ANMOL’s implementation challenges were linked to what may have been poor initial fit with the population. A respondent felt that although ‘as an application it’s quite good’ it was ultimately made by ‘someone sitting in Delhi’ in order to generate better data for central decision making and analysis, not for the FLHWs who struggled with using smartphones and lower-level health system managers (KI09, government).

##### Evidence from research

Both technical partners and government officials suggested that evidence from research studies was less important to informing scale-up than showcasing feasibility (KI01, technical partner) or filling a clear need identified by government officials (KI06, donor).

It’s like, okay, so they [the government] had some evidence… But ultimately, they were interested in the fact that here’s a piece of software that people not only know how to use, but want to use, and are willing to pay for.” (KI01, technical partner)

All our cases were scaled and on track to be sustained, so ones with rigorous research showing impact (MA; precursors to CAS and TECHO+) did not appear to be better placed for long-term sustainability compared with cases with only anecdotal insight from implementation experiences (NCD app, ANMOL).

Nonetheless, respondents involved in CAS suggested that evidence played two positive roles in achieving scale-up. First, although ‘politics were far outweighing the evidence’ policy-makers did need some evidence of effectiveness in order to meet a requirement before going to scale (KI16, technology partner). Second, the process of evaluating a digital tool can improve it by cultivating a ‘learning culture’ (KI14, evaluator) among policy-makers, technology partners and implementors whereby actors sought data from baseline and process evaluation research to improve the programme.

#### Operationalisation

##### Rolling out use by FLHWs

While some tools like TECHO+made an effort to address what users at different levels needed and thus reportedly achieved high FLHW satisfaction, several tools initially struggled with FLHW engagement during implementation—however, these initial hiccups did not seem to affect the tools’ eventual scale-up. These challenges included some states failing to distribute advertisements although MA was supposed to be implemented alongside a media campaign explaining the course to FLHWs; and ANMOL being rolled out through trainings for 250–300 Auxiliary Nurse Midwives (ANMs) at a time, which was an unwieldy number of participants (KI05, technology partner/implementor).

Respondents had varied perspectives on whether to rapidly switch from paper registers to digital systems, or to have dual systems for a while before the transition to digital systems is complete. ANMs using ANMOL and TECHO+ are expected to continue maintaining their paper registers. The technological partner working on TECHO+ (KI13) explained that paper record keeping should be phased out gradually, and only after it is clear that the new digital system is working. Moreover, FLHWs are answerable to multiple actors beyond the health system, such as the district collector or the national census, who expect to refer to paper registers.

I completely don’t think it’s their fault because the demands of the health and nutrition programmes of data to be on paper is huge because it’s not just their programme who ask for the data, they have the census guys coming in and say hey show me the register. She can’t say I have everything on the phone I've stopped maintaining a register. (KI06, donor)

In contrast, the nutrition system, ICDS, decided to quickly switch Anganwadi workers (AWWs) from paper to digital record keeping through CAS in order to streamline front-line processes and avoid creating inconsistencies between the two data sources. Removing paper-based records was argued to increase CAS’s sustainability prospects, since it would be difficult to remove a digital system once the paper-based records were gone: ‘You will meet your goal if you get rid of paper registers’ (KI06, donor).

##### Procurement hurdles

Procurement, whether of handsets, maintenance contracts, server capacity, or platform services, emerged as a major challenge during the implementation process with implications for long-term sustainability. Procurement was not challenging due to financial shortages, but because government actors feared taking major contracting decisions (KI11, technology partner). Government actors are scrutinised when they issue large contracts and can be questioned about its transparency and appropriateness even years after signing off on procurement (KI11, technology partner). Nonetheless, a respondent (KI16, technology partner) also reflected that the converse of this reluctance was the speed of largescale procurement when a government actor identifies an opportunity to ‘get a piece’ of an expensive contract for personal benefit.

Programme developers and funders suggested that sustainability could be fostered by ensuring that government procurement started at the outset, rather than at the time of transition (KI02, technical partner; KI16, technical partner). This would ensure that procurement processes are developed long before handover and avoid creating an expectation that funders or technical partners would handle procurement over the long term.

#### Evolving stakeholder roles and relationships

##### Evolving roles in implementation

Once the decision to scale was approved, a tool’s success depended on the implementing organisation’s commitment and capacity in terms of project management, training and roll-out logistics, ongoing testing and improving of the tool and dedicated relationship building across actors including the government.

Despite being used by government, all cases were developed and implemented through extensive non-governmental inputs (table 1). In terms of sustainability, respondents reflected that although the most intense inputs are required early on, ongoing field presence is necessary to troubleshoot, upgrade software and provide sustained support to FLHWs. Planning for this handover to government was not clearly outlined for some digital tools, potentially creating a sustainability challenge. For example, the NCD app implementor (KI11) explained that his organisation did not plan to be involved ‘forever’ but lacked a realistic transition plan agreed by MoHFW. Although the government has developed institutions for managing digital systems and data, for example, National Informatics Centre, respondents diverged in whether they envisioned the government as likely to gain the capacity to effectively manage these solutions over the long-term, in terms of implementation, technological expertise and management, and strategic/technical inputs.

##### Donor and government funding commitments

Although donor contributions are not significant in terms of overall development funding in India, for all cases, donor commitment was key to scaling up digital tools for FLHWs because there is an ‘expensive curve’ at the beginning which requires external inputs (KI03, technical partner), and because donors can bring innovations in faster than the government, which in turn saves the government time and energy (KI06, donor). Respondents concurred that a digital tool must have a major donor offering to pay for initial phases. In fact, a respondent speculated that many programmes fail to move beyond the pilot phase because they lack sufficient external financial backing to spark government interest (KI03, technical partner).

In terms of sustainability, some respondents suggested an ideal transition arc, with donors taking the initial risk, demonstrating proof of concept, and then having the government take over. Furthermore, a few respondents said that in order to be sustainable, programmes needed to no longer be dependent on donor funding due to shifting donor priorities (KI11, implementor; KI03, technical partner; KI06, donor).

I think donors get bored… they want to go on to the next project. The Programme Officers are worried about their career, they're worried about the optics. […] Don't assume because you're doing good work you can get more [from the donor] … [Donors are] willing to go fund the next random country just because of politics and stars aligned with, you know, what was a priority at the [donor’s], what was the priority for that Minister, and a conversation had happened (KI16, technical partner)

However, other respondents did not consider government funding as a golden bullet to sustaining digital tools for FLHWs, as even with government funding, programmes could still be cancelled (KI01, technical partner). Furthermore, several respondents did not consider sustainability in light of donor versus governmental funding (KI09, government; KI02, technical partner).

Beyond the source of funding, respondents noted examples of programmatic adaptations made to conserve finite resources. For direct-to-FLHW solutions like MA, to limit the risk of unplanned and unaffordable call costs generated by non-FLHWs, the programme used an approach called ‘white-listing’. Only FLHW numbers registered in government tracking databases were allowed to access MA. Any number that called MA that was not registered would hear an audio message indicating that the programme was for FLHWs and if the number had been changed, they should register the new number to receive access to the service.

### Context

Data from key informants highlighted national-state dynamics and ministry and programme fragmentation leading to interoperability challenges as influential for scaling and sustaining digital tools.

#### National-state dynamics

India’s federated structure generates a tension between national- and state-level decision-making, ownership and control. There is no standardised model for national- to state-level scaling up. When the national government decides to scale a digital tool, they offer it to states alongside funding to enable uptake.

This federal structure limited scale-up of the NCD app to a certain extent because states with higher capacity and their own state-level systems (eg, Tamil Nadu, Kerala) were hesitant to switch to the new national government-supported programme (KI11, technology partner). In contrast, TECHO+ was solely developed in Gujarat state with extremely strong state buy-in, but no national profile. A national government actor (KI12) lamented the missed opportunity of expanding TECHO+, explaining that Gujarat created a ‘beautiful’ application but since it only works within Gujarat’s Reproductive Child Health (RCH) health information system, it cannot be taken to other states.

Respondents did not agree on the ideal balance between national authority and state-level control. Some respondents wanted national government to take the lead in selecting tools because state governments ‘are so busy moving from one crisis to another’ (KI09, government) or because nationally standardised tools enable rapid and efficient implementation (KI06, donor). Others wanted the national government to provide leadership on setting standards and definitions for interoperability (eg, semantic interoperability to standardise the names for drugs) but to allow states to build solutions themselves. A senior national MoHFW official (KI12) felt that ‘innovation has to be everywhere’ and not only a national function; furthermore, national government should ‘create an ecosystem’ and standards within which states can innovate. If this standardisation is in place, each state can have its own system using a common application programming interface (API) and all the systems can speak to each other and state level data can be fed into a central database (KI01, technical partner).

#### Interoperability

The reality of disparate, vertical applications being implemented, often in response to government requests for a single-condition solution, was reported to impede sustainability and contribute to an environment of multiple solutions being implemented in overlapping geographies and across same FLHW cadres. Respondents noted that limited intersectoral collaboration between government ministries and within the health system resulted in the creation of parallel digital platforms that are not interoperable and entrench a siloed approach to the delivery of healthcare, nutrition and other social services. The challenge of interoperability between digital tools within the MoHFW is enormous, but even if they all aligned with the underlying RCH platform, there are multiple other ministries building digital tools that are not interoperable with RCH (KI03, KI04, technology partners).

The most striking example of ministry fragmentation can be found with CAS in the MoWCD and multiple tools in the MoHFW. At the village level, AWWs use CAS, and interact regularly with ANMs, who use ANMOL, TECHO+ and/or the NCD app. Notably, CAS operates on Dimagi’s CommCare platform, which does not integrate with RCH, which is the platform with which the ANM’s digital tools interoperate.

If ICDS-CAS was interoperable then you would be able to integrate the data you have collected from ICDS-CAS with [RCH]. We are talking about probably the same beneficiaries in two different systems […] which is not great (KI04, technology partner)

While the digital tools serve the same communities and same people, particularly pregnant and postpartum women and children, they require FLHWs from two different programmes (nutrition and health) to separately enumerate beneficiaries and collect similar datasets about them. Moreover, malnutrition or health issues identified by FLHWs in one programme and flagged through one digital tool will not integrate on the other tool, thus institutionalising gaps. One respondent (KI14, evaluator) noted that despite this fragmentation arising from the separate government ministries, FLHWs themselves often collaborated, and several states showcased positive collaboration. Respondents who had participated in efforts to integrate CAS and RCH were pessimistic about resolving national-level fragmentation because of ‘egos’ (KI14) and ‘turf protection’ (KI16). They also explained that in the interest of individual departmental needs and requirements, it appeared that both Ministries wanted to subsume the other’s digital system.

## Discussion

Given that so few digital programmes scale and then sustain, our study examined several scaled digital tools for FLHWs in India to understand both scale and sustainability of digital health programmes. While digital programmes are assumed to be amenable to scale due to the promise of technological innovation, we found not only technical complexity of digital tools, but also significant systems or relationship complexity, requiring iterative governance approaches to relationship building.

Frameworks for scaling up interventions consider the nature of the intervention and its delivery strategy, attributes of the actors involved and elements of the sociopolitical context.^8^ Studies of scaling up interventions concur in their conclusions about the unpredictability of the process and the concerted relationship building involved. The technological complexity of digital health programmes further amplifies the importance of such conclusions, particularly because they are not automated solutions, but require significant technological, social service delivery and governance adjustments.^13 32 33 36^ Not only do they require ongoing support from stakeholders outside the health system (technology partners) whose motivations or incentives may or may not align with health system actors, but they must overcome implementation challenges that are unique to digital tools (interoperability, data governance, ongoing innovation and technological support).^43^

While factors enabling scale and sustainability did overlap, respondents also noted several nuances that distinguished between them (table 3). Respondents emphatically noted the importance of state-level and national-level policy champions for successfully scaling digital tools; however, sustainable innovations also need long-term sustainable support and buy-in across levels and actors, especially in government. These cases also illustrate the importance of committed teams and strong leaders across donor, technology, technical and implementing organisations. As policy champions are political actors who are often reappointed to new positions or leave government if there is a change in party leadership, to be sustainable, digital tools need strategies to engage a churning succession of leaders and a mechanism to build and sustain relationships with bureaucrats below the policy champions.^44 45^

**Table 3:** Factors influencing scale and sustainability of digital tools for front-line health workers (FLHW) in India

Factors	Scale	Sustainability
Digital health solution characteristics		
Perceived value	The solution responds to the needs of various actors. Example: FLHW data capture and decision-support solutions streamline data collection systems and improve the timeliness, quality, accessibility and use of data. Direct to FLHW solutions affordable, standardised and logistically simple mechanism to refresh face to face training and fill in knowledge gaps.	Institutionalises support, supervision, and performance monitoring. For FLHW data capture and decision-support tools, the solution should move beyond data capture for report generation to use of data at multiple levels of the health system to improve provider performance and quality of service delivery.
Adaptability	Configurable software is seen as a more scalable approach which can accommodate differences in state-level health programmes and priorities. Extensibility is driven by infrastructure and telecommunications connectivity choices.	Desired configurability to allow for changes over time (eg, addition of new curriculum for Mobile Academy; NCD content added to TECHO+).
Data storage and governance	Use of high-quality cloud-based data storage (eg, Mobile Academy). Features of data governance, including data privacy, access, consent, not considered to be a key enabler of scale. Failure to prioritise data governance features have likely had adverse consequences on consent capture at the front lines.	Data governance largely not considered, but understood by some as concerning. Evolving data protection legislation is likely to have impact on data capture, procedural access controls, and consent processes.
Actor roles and relationships		
Government champions	Champions in influential government ministerial positions were vital to successful scale-up Communication between state government actors important Example: NCD app and Mobile Academy were first implemented in one state with support from senior actors in that state’s government, who praised the programme when interacting with other states, thus creating champions in other states.	Sustained engagement from influential government actors critical. The movement (due to transfer, retirement, other) of these champions is a significant barrier to sustainability. NDHM would also be a factor in enabling sustainability—so not an individual champion but a government champion if you will.
Stakeholder networks	Important to scaling digital tools for FLHWs Example: A government champion pushing CAS forward to approve decision to scale.	Argued as key to longevity of digital tools for FLHWs Example: For Mobile Academy, importance of cultivating deeper relationships going down to all implementers/FLHWs and broader buy-in to ensure the programme continues when there is turnover of key individuals. Or the NCD one that allows for adding other services.
FLHW engagement	FLHWs are supported to use technologies	Continuous FLHW engagement and feedback is integral to the longevity of the digital tool Example: FLHWs value and demand the tool, and want it fixed if it breaks or crashes.
Implementation processes		
Investing in evidence	Formative research used to design solutions Example: Some cases underwent extensive user testing and embedded development (MA, NCD app, TECHO+), while others were first developed by technology partners with specifications/data capture features stipulated, and then adjusted when implemented (ANMOL).	Evidence linking digital solutions to changes in health outcomes (impact) reported to be desirable. Routine use of system generated data seen as integral to demand creation and learning from evidence.
Operationalisation	Programme roll-out fosters FLHW engagement and ensures digital tool addresses FLHW needs Varied perspectives on whether to discontinue use of paper records immediately, or in phases. Procurement processes are initiated at the outset of the programme versus at the point of transition to government.	Programme roll-out fosters FLHW engagement and ensures digital tool addresses FLHW needs Procurement processes are initiated at the outset of the programme versus at the point of transition to government.
Evolving stakeholder roles and relationships	Initial donor investment integral to enabling scale. Programmatic adaptations may be required to conserve finite resources.	Concerns about government capacity to ensure handovers. Government funding is important but not necessarily sufficient for sustainability, as even with government funding, programmes could still be cancelled.
Context		
National-state dynamics	Standardised model for national-level to state-level scaling up does not exist. Several notable examples of state-level solutions which have scaled. National government leadership in establishing standards for interoperability.	Requires more robust data governance in long term Example: The national government could create an ecosystem and standards within which states can innovate. If this standardisation is in place, each state can have its own system using a common API and all the systems can speak to each other and state level data can be fed into a central database.
Interoperability	Intersectoral collaboration between government ministries required to reach agreement on common architecture and standards for interoperability.	Intersectoral collaboration between government ministries required to reach agreement on common architecture and standards for interoperability. Need to mandate adherence to standards once agreed on Example: It is important to ensure that all digital platforms built by all Ministries are interoperable with each other to eradicate siloed approaches to the delivery of healthcare, nutrition and other social services.

NCD, non-communicable diseases; NDHM, National Digital Health Mission.

For all cases, we found that genuinely productive partnerships with the potential to sustain digital solutions require working with ministries that ‘put some money on the table’ from the beginning, along with a major donor offering to pay for initial phases (KI06, donor). Once the decision to scale was approved, success depended on the implementing organisation’s commitment and capacity in terms of project management, training and roll-out logistics, ongoing testing and improving of the tool, and dedicated relationship building across actors including the government. Those seeking to develop, scale and sustain digital tools should note the two central themes related to partnership engagement: the importance of investing in relationships and the power of communication between actors within government. These findings are similar to other studies examining national scale-up that found invoking policy champions and building implementer capacity are key ingredients to catalysing scale-up.^10 46 47^

Planning for handover to government needs to be clearly outlined. Procurement, whether of handsets, maintenance contracts, server capacity or platform services, emerged as a major challenge during the implementation process with implications for long-term sustainability. Even once a tool is successfully scaled-up, the handover to government management can stall, not due to cost but because of procurement, which was challenging not due to funding shortages, but because government actors were afraid of taking major contracting decisions. These challenges are not only faced in more resource-constrained settings, as Lennon *et al* report similar red tape and ‘procurement pains’ when evaluating a digital health innovation programme in the UK.^32^

Data highlighted the following components of the government health system context as particularly important for sustaining digital tools: government capacity, dynamics between the national and state levels, and intersectoral collaboration. Siloed approaches to the roll-out of the studied digital solutions leading to interoperability challenges, among others, echo findings from a systematic review of mHealth and telehealth interventions in India that reported most of their included interventions were implemented as standalone solutions, often with no health systems integration strategy.^48^ It is thus promising to see the recent finalisation of National Digital Health Blueprint (NDHB), which, when implemented, will enforce standards that will allow for seamless interoperability.^49^ Furthermore, the NDHB will redress data architecture and governance challenges reported in our study and implement some key recommendations made by Tiffin *et al*^50^ to reduce the risks of health data breach or misuse in digital health programmes in low-income and middle-income countries, by creating a multitier system that ensures the highest levels of data security and ensuring privacy with Indian citizens as the owners of their data.

### Limitations

First, we studied scaled programmes that are in the midst of their implementation, thus our findings, to a certain extent, speculate on what will facilitate or hinder sustainability of these digital tools in the Indian health programming landscape. This means that while the factors that facilitated scale-up (in terms of the digital solution, actor roles and relationships, implementation processes and context) are based on respondent observation of actual practice, their implication on sustainability is speculative and requires longitudinal study. This is a noted limitation in many sustainability studies which do not have longitudinal or extensive retrospective designs.^12 15 26 51 52^ Second, given our emphasis on understanding policy design decisions rather than implementation experiences, we did not include FLHWs among respondents. Additional research to capture front-line experiences of the digital tool, the scale-up process and the actors involved, as well as their views on sustainability would enrich the story. Third, due to the nature of qualitative research and the limited time available from senior government officials, some components of our framework were not evenly probed in each interview, which may have hindered the comparability of the digital tools studied. Finally, the COVID-19 pandemic impacted the availability of some identified respondents, including additional donors, to be interviewed, which led to a smaller sample size in our study than we had anticipated, potentially reducing the robustness of the case studies.

## Conclusion

Digital innovations, despite their potential for scale, are not scaled or sustained in either an automated or linear manner. Donors, tool developers, implementors and government actors seeking to scale and sustain digital tools must focus not only on technical capacity but also on investing in people, relationships and service delivery adjustments to overcome significant challenges and uncertainties. It is imperative that we learn from the complexities involved to realise the potential promises of digital health.

## Data Availability

Data are available on request. Deidentified participant data are available on request from NSS (neha.singh@lshtm.ac.uk).
